# Supplementary material for: Intercorrelation of Molecular Biomarkers and Clinical Phenotype Measures in Fragile X Syndrome
Source: Cells. 2023 Jul 24;12(14):1920. doi: 10.3390/cells12141920 (PMC10377864; doi:10.3390/cells12141920)
Supplement: Supplementary file 1 [file cells-12-01920-s001.zip › cells-2430949-supplementary.pdf]

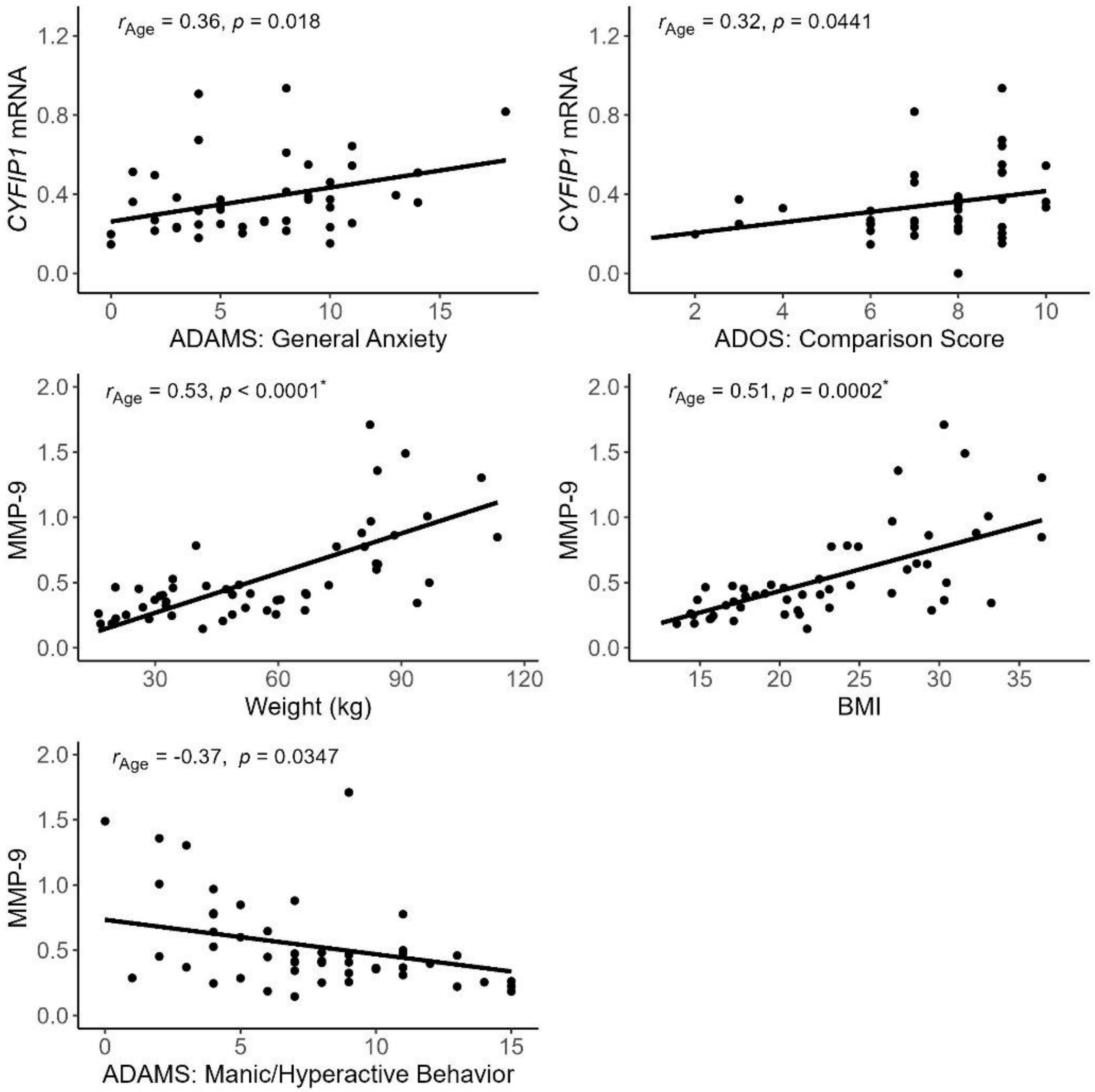

Supplemental Figure S1. Scatter plots of relationships of *CYFIP1* mRNA and MMP-9 with selected clinical measures.

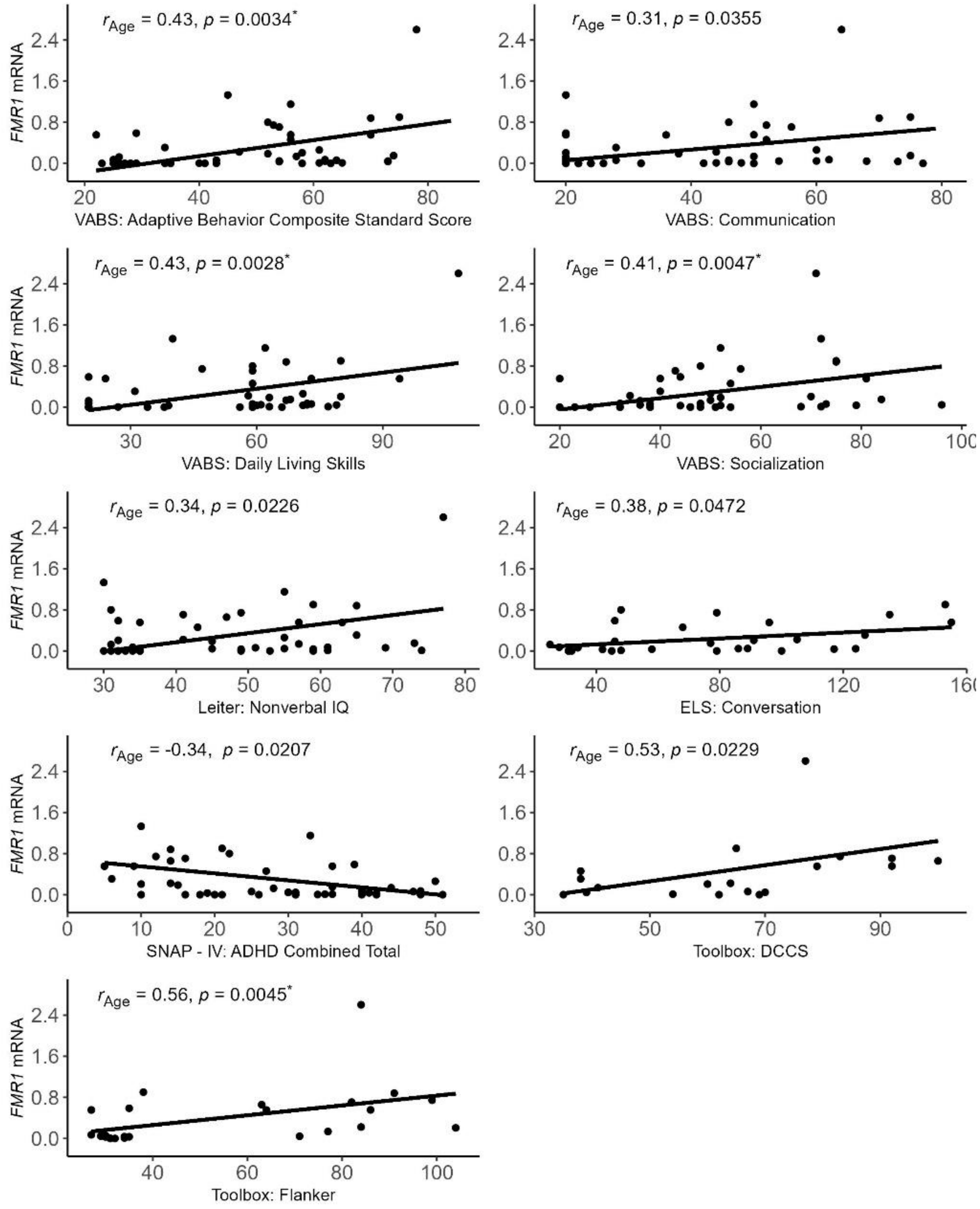

Supplemental Figure S2. Scatter plots of relationships of *FMR1* mRNA with selected clinical measures.
